# The cognitive-behavioral system of leadership: cognitive antecedents of active and passive leadership behaviors

**DOI:** 10.3389/fpsyg.2015.01344

**Published:** 2015-09-07

**Authors:** Edina Dóci, Jeroen Stouten, Joeri Hofmans

**Affiliations:** ^1^Faculty of Psychology and Educational Sciences, Vrije Universiteit BrusselBrussel, Belgium; ^2^Department of Psychology, University of LeuvenLeuven, Belgium

**Keywords:** transformational leadership, transactional leadership, core self-evaluations, cognitive antecedents of leadership, leaders’ beliefs

## Abstract

In the present paper, we propose a cognitive-behavioral understanding of active and passive leadership. Building on core evaluations theory, we offer a model that explains the emergence of leaders’ active and passive behaviors, thereby predicting stable, inter-individual, as well as variable, intra-individual differences in both types of leadership behavior. We explain leaders’ stable behavioral tendencies by their fundamental beliefs about themselves, others, and the world (core evaluations), while their variable, momentary behaviors are explained by the leaders’ momentary appraisals of themselves, others, and the world (specific evaluations). By introducing interactions between the situation the leader enters, the leader’s beliefs, appraisals, and behavior, we propose a comprehensive system of cognitive mechanisms that underlie active and passive leadership behavior.

## Introduction

The Full Range of Leadership Model portrays leadership as a pool of behaviors, ranging from highly passive to highly active (Avolio and Bass, 2001, p. 4; Avolio, 2010, p. 66; Sosik and Jung, 2011, p. 9). The model suggests that all leaders display both active and passive leadership, but that they do so with different frequency. Thus, some leaders have a stronger tendency to engage in active behaviors, while others are more likely to act passively. However, the Full Range of Leadership Model – or any other theory for that matter – falls short of explaining *why* some leaders are inclined to engage in active and others in passive behaviors, in other words, what the cognitive antecedents are that explain the differences in active and passive leadership behavior between different individuals. Furthermore, it also fails to clarify why the same leader would behave more actively in one situation than in another. Answering both questions is of particular importance for the leadership domain, both from a theoretical and a practical point of view.

Whereas researchers have in the last decades set out to examine dispositional, person-related antecedents of the full range of leadership behaviors (Barling et al., 2000; Judge and Bono, 2000; Peterson et al., 2009; Resick et al., 2009), surprisingly little attention has been paid to studying the antecedents of their within-person fluctuations (Nielsen and Cleal, 2011). Because relationships that exist at the between-person level do not necessarily apply to the within-person level (Hamaker, 2012), theorizing about and studying both between- and within-leader differences is crucial to arrive at a solid understanding of leadership behavior. Provided that intra-individual fluctuations in behaviors supplement inter-individual fluctuations (Tett and Guterman, 2000; Fleeson, 2001; Marshall and Brown, 2006; McNiel and Fleeson, 2006), there is a need for leadership theory that integrates intra-personal dynamism with stability, thereby conceptualizing them as two facets of the same phenomenon.

Furthermore, in recent decades researchers have accumulated a substantial amount of evidence showing the positive effects of active, transformational leadership behaviors on employee well-being and work outcomes (Bass, 1996, 1997; Yukl, 1999; Bass et al., 2003; Skogstad et al., 2007). Promoting active leadership behaviors among leaders is therefore highly beneficial both for individuals and organizations. However, solely knowing which dispositions go hand in hand with active leadership behaviors does not offer a sufficient theoretical basis for effective intervention. Only by incorporating intra-individual variation in leadership behavior into the scope of investigation, and by identifying the cognitive mechanisms that trigger favorable leadership behaviors, it is possible to offer relevant contributions for practitioners. We suggest this for a simple reason: it is easier for leaders to alter the way they think when it interferes with effective behaviors than to change their ‘unhelpful’ dispositions. Furthermore, to date most training programs merely teach new behaviors to leaders. However, if the cognitive mechanisms that trigger leadership behaviors are overlooked, interventions can hardly have long-lasting effects (Emiliani, 2003), given that leaders’ impulses to think and act in a certain way are likely to override their fading memory of the learned behaviors (Day, 2001; Powell and Yalcin, 2010).

**FIGURE 1:**
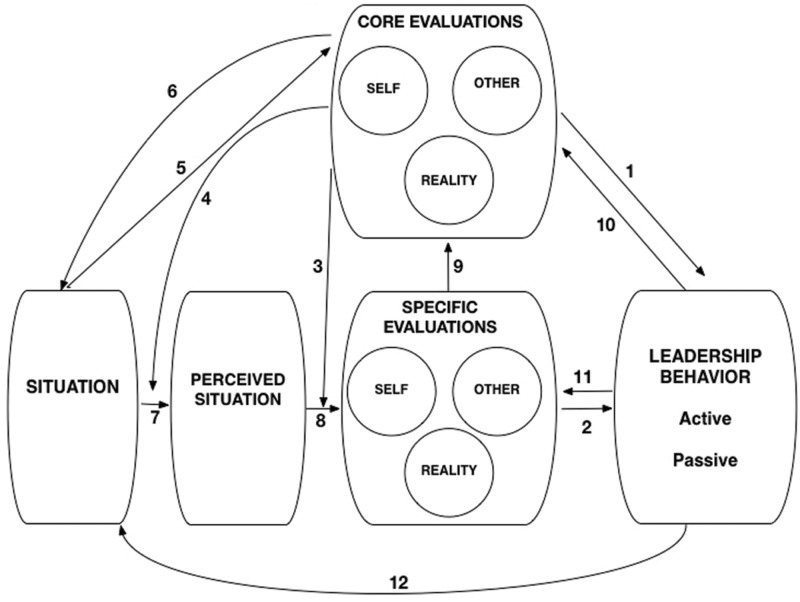
**The cognitive-behavioral system of leadership**.

In the present paper, we embark on offering theoretical answers to two questions that are crucial to improving our understanding of leadership behavior: (1) what are the cognitive antecedents that explain inter-individual differences in leadership behavior, and (2) what are the cognitive antecedents that explain intra-individual differences in leadership behavior. To this end, we propose the Cognitive-Behavioral System of Leadership (see **Figure 1**), a model that undertakes to explain both the stability and the dynamism in leaders’ behavior. To do so, we draw on one of the most influential interactionist theories, the Cognitive Affective Personality System (CAPS) theory (Mischel and Shoda, 1995). According to the CAPS theory, the same situational features are encoded differently in the minds of different individuals. For example, while an accounting task activates the encoding ‘easy’ for person A, it activates the encoding ‘difficult’ for person B. These encodings (e.g., *easy/difficult*) then activate other cognitive units, such as appraisals (e.g., *I can/cannot cope with this task*), beliefs (e.g., *I am a competent/incompetent person*), expectancies (e.g., *anticipating success/failure*), affects (e.g., *enthusiasm/anxiety*), goals (e.g., *problem solving/escaping*), and so on. Finally, a behavioral response (e.g., *proactive coping behavior/avoidance behavior*) is activated. The accessibility of certain cognitive units and the stable sequence of cognitive unit activation distinctive to a person is what accounts for the relative stability of behavior, while the different activation sequences set into motion by different situational features explain the flexibility in a person’s behavior (Mischel and Shoda, 1995; Schmitt et al., 2003). For example, in a socially (and not intellectually) challenging situation person B’s belief of incompetence does not get activated, and therefore s/he will engage in active rather than avoidance behaviors. The basic tenets of the CAPS model have already received support in the context of leadership theory, with Vroom and Jago (2007) suggesting that the stable sequences of cognitive unit activation are responsible for the patterns of variability in the actions, thoughts, and feelings of leaders across different situations.

**Table:** 

**Proposition**
(1) Core self-evaluations and leadership style	Arrow 1	Leader’s core self-evaluations positively predict active and negatively predict passive leadership, such that the more positive a leader’s core self-evaluations are, the more frequently s/he will engage in active and the less frequently in passive leadership behaviors.
(2) Specific self-evaluations and leadership behavior	Arrow 2	Leaders’ specific self-evaluations positively predict active and negatively predict passive leadership behaviors, such that the more positive a leader’s specific self-evaluations are in a situation, the more likely that s/he will display active and the less likely s/he will display passive leadership behaviors.
(3) Core other-evaluations and leadership style	Arrow 1	Leaders’ core other-evaluations positively predict active and negatively predict passive leadership, such that the more positive a leader’s core other-evaluations are, the more frequently s/he will engage in active and less frequently in passive leadership behaviors.
(4) Specific other-evaluations and leadership behavior	Arrow 2	Leaders’ specific other-evaluations positively predict active and negatively predict passive leadership behaviors, such that the more positive a leader’s specific other-evaluations are in a situation, the more likely that s/he will display active and the less likely s/he will display passive leadership behaviors.
(5) Core world-evaluations and leadership style	Arrow 1	Leaders’ core world-evaluations positively predict active and negatively predict passive leadership, such that the more positive a leader’s core world-evaluations are, the more frequently s/he will engage in active and less frequently in passive leadership behaviors.
(6) Specific world-evaluations and leadership behavior	Arrow 2	Leaders’ specific world-evaluations positively predict active and negatively predict passive leadership behaviors, such that the more positive a leader’s specific world-evaluations are in a situation, the more likely that s/he will display active and the less likely s/he will display passive leadership behaviors.
(7) The cognitive conditions of active leadership		Leaders’ self-, other-, and world-evaluations (both on the trait and state level) will interact, such that they will amplify each other’s positive effect when predicting active leadership.
(8) The emergence of specific evaluations	Arrow 3	Leaders’ core evaluations will moderate the relationship between the situational features and specific evaluations.
(9) Core evaluations and objective and perceived situation	Arrow 4	Leaders’ core evaluations will moderate the relationship between objective situational features and perceived situational features
(10) Situations solidify/modify core evaluations	Arrow 5	Situational features to which the leader is frequently exposed shape the leader’s core evaluations over the long term, such that (1) when the two are aligned (e.g., supportive organization and positive core evaluations), frequently experienced situations reinforce core evaluations, and (2) when the two are non-aligned (e.g., hostile organization and positive core evaluations), situations may modify core evaluations.
(11) Core evaluations and situation selection	Arrow 6	The more positive a leader’s core evaluations are, the more likely s/he will enter – socially or intellectually – challenging situations (while the more negative the leaders’ core evaluations are, the more likely s/he will avoid such situations).
(12) Specific evaluations solidify/modify core evaluations	Arrow 9	The leader’s frequently repeated specific evaluations shape the leader’s core evaluations over the long term, such that (1) when the two are aligned, specific evaluations reinforce core evaluations, and (2) when the two are non-aligned, specific evaluations may modify core evaluations
(13) Situations shape specific evaluations	Arrows 7–9	Situations shape the leader’s core evaluations by (re)shaping the leader’s specific evaluations.
(14) Leadership behaviors shape core evaluations	Arrow 10	The leader’s recurrent behaviors shape the leader’s core evaluations over the long term, such that (1) when the two are aligned with each other (positive core evaluations and active leadership behaviors, or negative core evaluations and passive behaviors), behaviors reinforce core evaluations, and (2) when the two are non-aligned, behaviors may modify core evaluations.
(15) Leadership behaviors shape situational features and specific evaluations	Arrows 5 and 12; Arrows 9 and 10	The leader’s behavior shapes the leader’s core evaluations through (1) modifying the situational features, and (2) modifying the leader’s specific evaluations

In the present article, we focus on two types of cognitive units drawn from different levels of a leader’s cognitive map. First, building on the core evaluations theory (Judge et al., 1997), we look at leaders’ fundamental beliefs about themselves, others, and their environment. These *core evaluations* are cognitive units that are relatively stable within one person, and affect every other unit above them. We suggest that, because they influence all appraisals, expectancies, and behavioral scripts, they shape leaders’ trait-like, dispositional tendencies for leadership behavior. Second, we turn our attention to leaders’ appraisals of the self, others, and the environment. These *specific evaluations* vary over time and between different circumstances for one leader, as they are triggered by a particular context. As such, they can explain the fluctuating, state-like dynamics in a leader’s behavior. Together, core evaluations and specific evaluations help to explain the interplay of stability *and* variability in leadership behavior.

In the analysis below, the role of core evaluations and specific evaluations in the emergence of leadership behavior is discussed in more detail. Subsequently, discussion moves onto the dynamic interplay between the stable trait and the variable state level of our model, and the interactions between the situation, the leader’s core evaluations, specific evaluations, and behavior. Besides proposing original relationships, we also aim to incorporate diverse, existing knowledge about the antecedents of leadership behavior into one coherent model. Therefore, some of the suggested relationships do not have great novelty value of themselves.

## The Full Range of Leadership Model

According to the Full Range of Leadership Model (Avolio and Bass, 1991), at the passive end of the leadership spectrum lies the *lack of leadership*. *Laissez faire* is the management style characterized by the avoidance of taking leadership responsibilities, decisions, and actions, even in dire circumstances. Moving further along the passive-active continuum, the next leadership behavior is *passive management by exception.* This term refers to a management style whereby the leader does not act until problems get out of hand. Next on the scale is *active management by exception*, a management style characterized by looking out for mistakes, problems, and violations of the rules, and monitoring, controlling, and disciplining subordinates. This leadership style is active in the sense that the leader takes control of the situation instead of the situation controlling the leader. The leader actively monitors the deeds of the subordinate and intervenes when the subordinate’s performance does not live up to expected standards. Even though active management by exception is clearly an active way of managing in comparison to laissez faire and passive management by exception, it is nevertheless a reactive rather than proactive way of leading. Therefore its midway position on the passivity-activity continuum. Sitting further toward the active end of the scale is *contingent reward* leadership, whereby reward are made contingent on subordinates’ performance, and the necessary steps in order to be rewarded are stipulated. Contingent reward leadership implies a higher level of activeness and initiation than the previous behaviors, as the leader sets goals, identifies objectives, and structures expectations. However, a considerable amount of energy is still spent on ‘reacting,’ following up on objectives, rewarding, and punishing subordinates. Management by exception behaviors and contingent reward leadership fall under the umbrella term ‘transactional leadership.’ In all transactional leadership behaviors the leader-subordinate relationship is essentially based on the principle of mutually self-interested exchange. Transactional leaders’ schema of leadership suggests that employees get their work done in exchange for financial and other reward from their leaders. Besides rewarding them, transactional leaders ‘motivate’ their subordinates by monitoring, controlling, and punishing them (Bass, 1991, 1999; Barling et al., 2011).

*Transformational leadership*, on the other hand, is based on the leader’s ability to motivate subordinates by providing them with an appealing vision and stimulating challenges, and by being an inspiring role model. The four transformational behaviors, which highly intercorrelate (Avolio et al., 1999), constitute the active end of the leadership spectrum. *Individualized consideration* refers to the extent to which the leader attends to each subordinate’s needs, provides them with empathy and compassion, listens to them actively, recognizes their strengths, and helps to develop their skills by acting as a mentor or coach. *Intellectual stimulation* refers to the extent to which the leader challenges the subordinates’ beliefs and assumptions, takes risks, and promotes independent thinking on the part of subordinates. *Inspirational motivation* refers to the extent to which the leader articulates an inspiring vision, creates a sense of purpose and provides the subordinates with high but realistic standards. *Idealized influence* refers to the extent to which the leader acts with integrity and as a role model for high ethical behavior, is driven by what is best for the subordinates and the organization, stands up for her/his values, addresses crises head-on, and acts charismatically (Bass, 1999; Kirkbride, 2006; Barling et al., 2011). (Because of their high intercorrelations, throughout the article we discuss the four transformational behaviors collectively, as facets of the same leadership style.) In sum, transformational leadership is characterized by highly (pro)active behaviors, such as innovating, risk-taking and challenging others, elevating expectations, shaping meaning and creating purpose.

As mentioned above, transactional leadership can also take on active forms, as the name ‘active management by exception’ indicates. Indeed, it requires a considerable level of alertness and action-orientation from the leader to closely monitor subordinates and maintain an early warning system should mistakes arise (Kirkbride, 2006). The Full Range of Leadership Model suggests that it is nevertheless a less active form of leading than transformational behaviors. The reason for this is that even though active management by exception entails attention, vigilance and action, it is nevertheless reactive in the sense that the leader attends mainly to deviations and corrects subordinates’ behaviors when performance deteriorates from required standards (Bass, 1997; Avolio et al., 1999). Transformational leadership behaviors, instead, are approach-oriented, initiating, pro-active ways of leading, whereby the leader inspires and stimulates subordinates to reach more than they thought was possible.

## Explaining Stable, Inter-Individual Differences in Leadership Behavior: Core Evaluations

We propose that, in order to understand leaders’ dispositional tendencies to display active or passive leadership behavior, we must identify their core beliefs: their deepest, most enduring understandings about the self, others, and the world (Beck, 1967, 2011). The reason is that core beliefs are deep cognitive structures that guide the selection, encoding, and evaluation of all stimuli (Beck, 1967), with a major impact on subsequent behavior (Segal, 1988). In other words, beliefs influence the formation of appraisals, which in turn activate behaviors. For example, if a person believes that the world is an unfair place, s/he may perceive any criticism as hostile, and as a result act vengefully when being criticized. Identifying a leader’s underlying beliefs therefore provides insight into the relative stability in his/her cognitive and behavioral patterns (McGregor, 1960; Krishnan, 2001; Emiliani, 2003; Tickle et al., 2005; Washington et al., 2006; Pastor and Mayo, 2008).

People hold beliefs about every segment of life (e.g., women are gentle, traveling is exciting, learning is difficult, etc.). However, according to *core evaluations theory* (Judge et al., 1997), there are only a small number of fundamental beliefs that underlie every subsequent appraisal. Such beliefs are referred to as *core evaluations*, that is, bottom-line, all-encompassing, and evaluative beliefs that an individual holds. Core evaluations comprise three areas, namely the self, other people, and the world (Judge et al., 1997*).* In this article, we argue that individual differences in the three types of core evaluations (i.e., core self-evaluations, core other-evaluations, and core world-evaluations) account for individual differences in leaders’ inclinations to practice active or passive leadership behaviors (**Figure 1**, arrow 1). For example, leaders who fundamentally trust the world to be a safe place will generally be more inclined to take initiatives and risks than leaders who see the world as a dangerous place. Before elaborating on the relationship between leaders’ core evaluations and behavioral tendencies, we will introduce the idea that ‘specific evaluations’ of the self, others, and the world explain within-person fluctuations in leadership behavior.

## Explaining Intra-Individual Differences in Leadership Behavior: Specific Evaluations

Behaviors are not driven directly by the situation, but rather by the perceptions and interpretations of the situation by the subject (Lewin, 1951; Weiner, 1980; Ajzen, 1991; Mischel and Shoda, 1995; Kuppens et al., 2009). Building on this premise, we suggest that momentary leadership behavior results from the meaning that the leader attributes to any situation, that is, how s/he perceives him/herself, others, and the environment in a certain context. Such appraisals are the leader’s *state core evaluations* or *specific evaluations* that – as opposed to core evaluations – fluctuate across situations (for state core self-evaluations, see Judge and Kammeyer-Mueller, 2011; Nübold et al., 2013).

Specific evaluations fluctuate because situations change. In some situations Leader A feels that s/he can cope well with the demands of the situation, while in others s/he may experience a lesser degree of confidence and control. Sometimes Leader A finds the subordinate s/he is interacting with reliable, while at other times s/he perceives the subordinate to be unreliable. Even if Leader A usually appraises the organizational environment to be safe and just, occasionally s/he may perceive it as threatening and unfair. We suggest that such different appraisals will trigger different behavioral responses within the same leader (**Figure 1**, arrow 2). For example, when a leader feels capable of coping with a task’s demands, s/he may also be capable of actively inspiring and challenging others, while in a situation where s/he does not feel in control of the situation, s/he may be inclined to remain passive.

## Core Evaluations, Specific Evaluations and Leadership Behavior

### Core Self-Evaluations and Leadership

Core self-evaluations are the fundamental evaluations an individual holds about him/herself and her/his capabilities, self-worth, and ability to cope (Judge et al., 1997). It is a higher-order trait indicated by four lower-order traits: locus of control, generalized self-efficacy, self-esteem, and neuroticism. Locus of control refers to a person’s belief about the causes of events in his/her life. People with an internal locus of control believe that they shape the events in their lives, while people with an external locus of control attribute the causes of events to external factors such as luck or other people’s actions (Rotter, 1966). Generalized self-efficacy refers to a person’s beliefs about being able to cope successfully with a wide range of life-situations (Smith, 1989). Self-esteem refers to a person’s self-acceptance, self-liking and self-respect (Judge et al., 1997), and neuroticism refers to one’s tendency to experience long-lasting, negative emotions (Costa and McCrae, 1992).

Resick et al. (2009) have found that leaders with positive core self-evaluations are more likely to be transformational than leaders with negative core self-evaluations because they possess the necessary self-confidence required to perform transformational behaviors. People with negative core self-evaluations believe that they cannot cope successfully with challenging situations, and therefore they are inclined to engage in avoidance coping behaviors (Kammeyer-Mueller et al., 2009). We suggest that leaders who have strong inclinations to engage in passive forms of leadership – such as neglecting their responsibilities or avoiding action altogether – may do so because of their negative core self-evaluations.

Proposition 1: Leader’s core self-evaluations positively predict active and negatively predict passive leadership, such that the more positive a leader’s core self-evaluations are, the more frequently s/he will engage in active and the less frequently in passive leadership behaviors. (**Figure 1**, arrow 1).

#### Specific Self-Evaluations (State Core Self-Evaluations) and Leadership Behavior

Although core-self evaluations have predominantly been studied as a stable, person-related characteristic, there is by now widespread agreement that core self-evaluations should be seen as a trait- *and* state-based construct (Judge and Kammeyer-Mueller, 2004; Judge et al., 2012). This implies that a person’s core self-evaluations fluctuate (i.e., the state part) around a fixed point (i.e., the trait part). In line with this idea, recent research has demonstrated that one’s state core self-evaluations (*we use the terms state core self-evaluations and specific self-evaluations interchangeably*) indeed vary across situations (Debusscher et al., 2015; Dóci and Hofmans, 2015), just as has been shown regarding its constituent parts: self-esteem (Heatherton and Polivy, 1991), neuroticism (McNiel and Fleeson, 2006; Debusscher et al., 2014), and self-efficacy (Bandura, 2006).

As mentioned above, empirical evidence supports the notion that leaders with positive core self-evaluations are more likely to be predominantly transformational than leaders with negative core self-evaluations (Resick et al., 2009). We suggest that this relationship holds true on the state level too, that is, the more a leader feels in control, confident, and capable in a situation, the more likely s/he will challenge, inspire, stimulate, and coach others. Conversely, we also suggest that the less the leader feels in control and capable, the more likely s/he will display passive behaviors. An experimental study conducted by Dóci and Hofmans (2015) provided initial empirical evidence in support of this assumption by showing that state core-self evaluations were positively related to subordinate ratings of transformational leadership behavior.

Proposition 2: Leaders’ specific self-evaluations positively predict active and negatively predict passive leadership behaviors, such that the more positive a leader’s specific self-evaluations are in a situation, the more likely that s/he will display active and the less likely s/he will display passive leadership behaviors. (**Figure 1**, arrow 2).

#### The Criteria of Self-evaluations

From situation to situation, the criteria against which one evaluates oneself may differ. These criteria are dictated by the particular context, that is, the skills and competencies a certain situation requires. For example, while giving a presentation at a conference, one’s self-evaluations primarily depend on one’s appraisal of his/her scientific knowledge and presentation skills. In a dating situation, however, the same person’s self-evaluation may largely be a function of that person’s appraisal of her/his physical appearance. Thus, while the frame of reference for self-evaluations is ‘brains’ at a conference, it may be ‘looks’ in a dating situation.

When it comes to leadership, we suggest that there are two central domains in which one needs to feel capable, in order to arrive at positive self-evaluations (and engage in active behaviors): (1) handling people and (2) handling tasks. We expect these two dimensions of self-evaluations to be relatively independent from one another, meaning that one can evaluate oneself positively on one dimension and negatively on the other. For example, a manager at an airplane manufacturing plant with an organizational psychology background may feel confident about motivating his/her subordinates, but insecure when it comes to understanding the engineering problems at hand. Conceptualizing these two domains as distinct facets of self-evaluations is in line with research on the multi-faceted nature of self-esteem, that has shown that the sense of competence and the sense of social worth are two discrete dimensions (Tafarodi and Swann, 1995). Moreover, the two facets of self-evaluations we propose (handling tasks and handling people effectively) correspond to the two main behavioral requirements of the leadership role identified by the Ohio State Leadership Studies: *initiating structure* and *consideration*. These two criteria have been found to be independent from each other, so that the leader’s position on one dimension does not predict the leader’s position on the other dimension (Fleishman, 1953; Weissenberg and Kavanagh, 1972).

We suggest that for practicing highly active leadership behaviors, a leader must evaluate him/herself as capable in both domains. A leader needs to feel socially confident to coach, stimulate, and inspire subordinates, and to act charismatically. Furthermore, the leader also needs to feel confident in relation to tasks in order to challenge assumptions, demonstrate competence, and offer innovative solutions. When the leader has low confidence in one or both domains, s/he may no longer be capable of displaying highly active, transformational leadership. Thus, we suggest that feeling confident about managing people *and* tasks are necessary (but not sufficient) pre-conditions for performing active leadership behaviors.

### Core Other-Evaluations and Leadership

Core evaluations of others (hereafter: core other-evaluations) refer to the implicit theory that an individual holds about other people, that is, whether others can generally be trusted (Judge et al., 1997). We propose that this fundamental belief plays a crucial role in leaders’ active behavioral inclinations. To be inclined to challenge, stimulate, inspire, and coach subordinates, a leader needs to believe that people are trustworthy, implying, in the leadership context, that people can be expected to fully discharge their work duties. Leaders who do not trust others may instead be prone to closely monitor subordinates and look out for mistakes. Furthermore, we suggest that leaders who have confidence in others may be more inclined to engage in interactions with their subordinates, as trust generates sociability (Fukuyama, 1995). Leaders who are apprehensive about others may instead be inclined to be socially passive and avoid interactions with their subordinates.

It has been found those leaders’ implicit followership theories, that is, their beliefs about what followers their perceptions are like in general, shape of and behaviors toward their subordinates (Goodwin et al., 2000; Sy, 2010). This line of research underscores the need for studying other-evaluations when trying to understand the differences between active and passive leadership behavior. Research that has shown that transformational and transactional leaders have distinct schemas about subordinates (Goodwin et al., 2000) points in the same direction. In particular, Pastor and Mayo (2008) found that transformational leaders were more likely to hold Y-beliefs than non-transformational leaders. Leaders with Y beliefs think that under the right circumstances, subordinates are reliable motivated to work and eager to take on responsibilities. Instead, leaders with X-beliefs see subordinates as inherently lazy and inclined to avoid tasks and responsibilities; therefore, they believe that subordinates must be closely monitored and controlled (McGregor, 1960). We suggest that Y beliefs are underpinned by a leader’s predisposition to evaluate people positively.

Proposition 3: Leaders’ core other-evaluations positively predict active and negatively predict passive leadership, such that the more positive a leader’s core other-evaluations are, the more frequently s/he will engage in active and less frequently in passive leadership behaviors. (**Figure 1**, arrow 1).

#### Specific Other-Evaluations and Leadership Behavior

In the previous section we discussed how core other-evaluations may incline leaders to perceive subordinates as generally trustworthy or untrustworthy. However, despite the existence of such a general tendency, people’s momentary level of trust varies as a function of who they are in interaction with (Mayer et al., 1995). Even though it has not yet been thoroughly examined in the framework of the Full Range of Leadership Model, extensive research has shown that leaders do change their behavior as a function of their perception of the subordinate (Lowin and Craig, 1968; Witkin et al., 1977; Turban and Jones, 1988). In particular, LMX theory claims that leaders change their behavior based on their evaluation of the abilities and attitudes of the different subordinates (Dansereau et al., 1975). When leaders trust their subordinates, they give them more time and attention, challenge them, and provide them with more opportunities to develop themselves than when they do not trust the subordinates (Graen and Uhl-Bien, 1995). Research has shown that when the level of trust toward a subordinate is low, leaders are more likely to emphasize their authority position and tighten control (Georgesen and Harris, 2006), intensify monitoring (Mayer and Gavin, 2005), and give subordinates less information, responsibility, and autonomy (Mayer et al., 1995).

Because trust is multidimensional and domain-specific (Lewicki et al., 1998), we suggest that the leader’s trust toward one particular subordinate also fluctuates. This fluctuation may be a function of the situation’s demands, and the leader’s evaluation of the subordinate’s capability and commitment to fulfill these demands. We predict that on occasions when the leader evaluates a particular subordinate positively, s/he will be inclined to perform active leadership behaviors characterized by pursuing contact with the subordinate, providing him/her with attention, support, stimulation, and inspiration. When the leader instead sees the same subordinate in a more negative light, s/he will be prone to emphasize her/his authority, tighten up control, intensify monitoring, or avoid the subordinate altogether. In sum, the more positively the leader evaluates a subordinate, the more inclined the leader will be to engage in active, and the less inclined to engage in passive leadership behaviors. As mentioned above, this proposition is closely aligned with the basic tenets of LMX theory. This is not a surprise given that active leadership behaviors have been shown to be closely associated with leader member exchange, as research has demonstrated that leader-member exchange acts as a mediator between transformational behaviors and positive work outcomes (Wang et al., 2005). This link implies that the same underlying cognitive mechanism may contribute to the emergence of both phenomena.

Proposition 4: Leaders’ specific other-evaluations positively predict active and negatively predict passive leadership behaviors, such that the more positive a leader’s specific other-evaluations are in a situation, the more likely that s/he will display active and the less likely s/he will display passive leadership behaviors. (**Figure 1**, arrow 2).

#### The Criteria of Other-Evaluations

Similarly to self-evaluations, we suggest that the evaluation of others is based on different criteria in different contexts. For example, in a hospital, person A (the patient) may find person B (the surgeon) trustworthy if person B has steady hands and a low mortality record. However, if A and B get married after the operation, fidelity may become the major criterion of B’s trustworthiness. Trust in another person is a belief that can be divided into several, independent components, so that the same individual can find one person (or group of people) trustworthy in one dimension, and untrustworthy in another (Mayer et al., 1995).

We suggest that for a leader to fully trust a subordinate, and therefore engage in highly active leadership behaviors; at least two criteria must be fulfilled. First, the leader must believe that the subordinate is *reliable* enough to perform his/her duties. Second, the leader must trust the subordinate to be *capable* of performing such duties, because even if the subordinate is willing to perform well, if s/he is not capable of doing so, convincing results cannot be realized. Obviously, a person can be perceived to be capable but not reliable, and vice versa.

Distinguishing between these trust domains corresponds to McAllister’s (1995) assertion that cognition-based trust is a function of the evaluation of the other person’s competence and reliability. We suggest that the leader needs to perceive the subordinate as both reliable *and* capable in order to perform highly active behaviors. To provide the subordinate with caring and emotional support, the leader must consider the subordinate reliable, and therefore worthy of a reciprocally positive attitude (*see Social Exchange Theory*, Emerson, 1976). Furthermore, to encourage the subordinate to think for him/herself, to stimulate and challenge him/her, and to solicit the subordinate’s ideas, the leader must trust the subordinate to be capable. If the leader evaluates the subordinate to be incapable and/or unreliable, the leader may instead be inclined to give clear instructions and follow up on them closely, to be on the lookout for mistakes and deviations, to closely control the subordinate, or to ignore him/her altogether.

The above-identified criteria for other-evaluations are closely in line with the situational leadership model (Hersey and Blanchard, 1969). The situational leadership model suggests that effective leaders must adapt their leadership behavior to the maturity level of the subordinate, which is represented by the subordinate’s level of competence and commitment. While the resemblance between the two models is obvious, they also differ in a core feature, namely that one of them describes the most fruitful, while the other describes the most probable leaderships behaviors in a situation. The situational leadership model is a contingency model that concerns the effectiveness of leadership behaviors in relation to subordinates with differing features, and describes the most functional and desirable managing style in particular circumstances. Our model, on the other hand, introduces the leadership behaviors that are the most likely to occur in certain circumstances. Thus, the two models firmly complement each other.

### Core World-Evaluations and Leadership

Core evaluations of the world (hereafter ‘core world-evaluations’) are an individual’s deeply held beliefs about the world around him/her (Judge et al., 1997), that is, whether the world can be trusted or not. The original core-evaluations theory distinguishes between three core world-evaluations: believing that the world is fundamentally benevolent (or malevolent); believing that the world is fundamentally just (or unjust); and believing that the world is fundamentally exciting (or dangerous) (Judge et al., 1997).

People who believe that the world is essentially benevolent trust their environment to be a safe and good place where success and happiness can be realized and values can be upheld (Judge et al., 1997, p. 164). As such, these people have the right disposition to engage in active behaviors, as research has shown that there is a higher chance for aspiration and goal-oriented action when one believes that success is likely (Jacobs et al., 1984; Wood and Bandura, 1989; Bandura and Locke, 2003). If a leader instead believes that the world is a bad place where success is an exception, values cannot be realized, and the rule is suffering and misery (Judge et al., 1997; p. 164), there is a good chance for withdrawal and passivity, based on an expectation of non-contingency between actions and probable future outcomes (see *learned helplessness theory*; Seligman, 1975; Maier and Seligman, 1976). If a leader thinks that it is not possible to achieve goals and values in this world, s/he may be less inclined to actively pursue them. In line with this idea, research has shown that not believing in the possibility for change in the organization negatively predicts transformational behavior (Bommer et al., 2004).

Furthermore, we suggest that leaders who believe that the world is an exciting place have the mindset that is needed to think innovatively, go on undiscovered paths, and challenge widely held beliefs, while leaders who think that the world is dangerous may not take the risks the aforementioned behaviors entail. Believing that the world is exciting rather than dangerous implies a generalized sense of psychological safety, and psychological safety has been shown to promote creativity in organizations (Edmondson, 2004), to relate positively to organizational innovativeness (Baer and Frese, 2003), and to promote information sharing in a way that inspires subordinates to develop their own creative ideas (Edmondson, 2004), all of which are features of active leadership. In line with this, the perception of a supportive and challenging – and therefore exciting rather than dangerous – organizational climate has been shown to promote high creativity (McLean, 2005, for a review). Moreover, believing that the world is a dangerous place implies a proclivity to fear, and fear has been shown to predict avoidance (Craske et al., 1987). Leaders who think that the world is dangerous may thus avoid taking risks in order to prevent getting harmed or punished in the event of failure, and rather withdraw into ‘safe’ passivity. Furthermore, leaders who believe that the world is a malicious place, instead of encouraging independent thinking may become controlling and hyper-vigilant for mistakes made under their supervision, in an attempt to pre-empt retaliation in what is perceived to be a hostile environment.

Proposition 5: Leaders’ core world-evaluations positively predict active and negatively predict passive leadership, such that the more positive a leader’s core world-evaluations are, the more frequently s/he will engage in active and less frequently in passive leadership behaviors. (**Figure 1**, arrow 1).

#### Specific World-Evaluations and Leadership Behavior

We suggest that the momentary appraisals a leader makes about the dangerousness, fairness, and benevolence of the environment in a particular situation shape the leader’s behavior in that situation. When the leader evaluates the environment positively (benevolent, just, exciting) s/he may be more inclined to challenge and stimulate others, be innovative and pursue change, than in situations when s/he appraises the environment negatively (malevolent, unjust, dangerous), inclining him/her to monitor and control subordinates or withdraw altogether.

Proposition 6: Leaders’ specific world-evaluations positively predict active and negatively predict passive leadership behaviors, such that the more positive a leader’s specific world-evaluations are in a situation, the more likely that s/he will display active and the less likely s/he will display passive leadership behaviors (**Figure 1**, arrow 2).

### The Cognitive Conditions of Active Leadership

We suggest that highly active, transformational leadership behaviors are most likely to emerge when all three cognitive conditions are met, that is, the leader evaluates him/herself, the subordinates, *and* the environment positively. For example, even if a leader has high confidence in him/herself and also appraises the organization to be fair and supportive, but thinks that the subordinate is not competent enough, s/he may not be inclined to engage in highly inspiring and challenging behaviors toward the subordinate. Similarly, even if the leader appraises the subordinate to be competent and the organization to be benevolent, but doesn’t have the self-confidence for the task at hand, s/he may not be able to engage in stimulating leadership. Finally, even if the leader believes that both him/herself *and* the subordinate are able to achieve excellent outcomes, but perceives the organizational environment as threatening, s/he may not be inclined to take risks and encourage innovativeness. Therefore, having positive evaluations of all three ‘actors’ provides a fertile cognitive ground for the emergence of active leadership behaviors. We suggest that negative evaluations (i.e., perceiving the self to be unable to cope, people to be untrustworthy or the context to be threatening) will instead activate defensive manoeuvers (Packer, 1985), such as avoidance behaviors or attempts at controlling others and the environment.

Proposition 7: leaders’ core self-, other-, and world-evaluations (both on the trait and state level) will interact, such that they will amplify each other’s positive effect when predicting active leadership.

## The Dynamic Interplay between the Situation, Core Evaluations, Specific Evaluations and Leadership Behavior

### The Emergence of Specific Evaluations

As argued above, different situations trigger different specific evaluations (**Figure 1**, arrows 7 and 8). For example, in a situation where subordinate X arrives late to a meeting, Leader A will evaluate X negatively, while in a situation where X arrives on time, Leader A may see X in a more positive light. However, specific evaluations are not only shaped by the situation, but rather by the interaction between the situation and the leader’s core evaluations (Kammeyer-Mueller et al., 2009). In the previous example, where subordinate X arrived late to a meeting (situational feature), Leader A, who holds the view that subordinates in general are unreliable (negative core other-evaluations), may think ‘He’s late because he couldn’t care less’ (negative specific other-evaluations). Leader B, however, who thinks that subordinates in general are reliable, may not attribute importance to being late, thus maintaining her/his positive image of X.

Proposition 8: Leaders’ core evaluations will moderate the relationship between the situational features and specific evaluations. (**Figure 1**, arrow 3).

### Interaction between Core Evaluations and the Situation

We propose that a leader’s core evaluations shape the leader’s perception of the situation. They predispose the leader to notice and magnify some features of the situation and ignore others, thereby attributing certain meanings to the features in such a way that they become aligned with the leader’s core evaluations (Kammeyer-Mueller et al., 2009). For example, leaders with negative core other evaluations may be prone to notice and magnify small deviations from the rules and perceive them as violations, while leaders with positive core other evaluations may not pay attention to such deviations. When subordinate X arrives 10 min late to the meeting, Leader A with positive core other-evaluations may think ‘X is a little bit late,’ while B, the leader with less confidence in others, may think ‘X is very late.’ In line with our reasoning, the *differential exposure hypothesis* suggests that people with positive core self-evaluations are less likely to interpret work situations as stressful as people with negative core self-evaluations (Kammeyer-Mueller et al., 2009). People with positive core self-evaluations also experience their job as more challenging because their positive predisposition makes them focus on the positive qualities of the job (Judge et al., 2000).

Proposition 9: Leaders’ core evaluations will moderate the relationship between objective situational features and perceived situational features. (**Figure 1**, arrow 4).

### Self-Preserving Mechanisms of the System

Human systems have their self-organizing dynamics and mechanisms to preserve themselves and their coherence (McGinn and Young, 1996). This, we suggest, holds true for the Cognitive-Behavioral System of Leadership too. In what follows, we will discuss the mechanisms through which the system remains self-preserving.

#### Situations Solidify Core Evaluations

Being exposed to certain working conditions over a long time period may lead to the maturation of core evaluations, as long-term working conditions have the potential to shape personality traits (Wille et al., 2013; Wille and De Fruyt, 2014). For example, the originally mildly negative core other-evaluations of Leader F may become absolute and incontestable after years of directing a school for dropouts, where students are often aggressive or absent, and teachers are cynical and negligent.

#### Specific Evaluations Solidify Core Evaluations

Repeated appraisals of the self, others, and the world (i.e., specific evaluations) across various situations cement the beliefs held about the self, others, and the world (i.e., core evaluations). This happens because repeated encodings increase the chronic accessibility of these cognitive units and make the neuron pathways become automatic (Mischel and Shoda, 1995). If the above-mentioned Leader F perceives students and teachers to be untrustworthy on a day-to-day basis, this will result in the solidification of her/his negative belief about people in general. Note that specific evaluations therefore mediate the link between the situation and core evaluations, a suggestion that is in line with the sociogenomic model of personality and its view of environments shaping personality traits by affecting states (Roberts and Jackson, 2008).

#### Leadership Behaviors Shape the Situation and Specific Evaluations and Solidify Core Evaluations

Another ‘tool’ for maintaining beliefs is behavior itself. Leaders preserve their beliefs by acting the way they do. The Cognitive-Behavioral System of Leadership is a self-reinforcing cycle in which the leader’s behaviors set positive and negative self-fulfilling prophecies into motion, that in turn validate the preconceptions that elicited the behaviors. This may happen through modifying the features of the context, as people tend to alter their environments to achieve consistency with their personality traits (Caspi et al., 2005). The modified context then provides the leader with further opportunities to collect evidence about the accuracy of his/her beliefs. For example, a leader with a generalized negative opinion about subordinates may engage in active management by exception behavior, focusing on followers’ mistakes and failures to meet standards. Consequently, subordinates may start to live up to the negative expectations (*Golem effect*; Eden, 1992), lower their efforts, and therefore confirm the leader’s negative ideas about subordinates. Consider also the *laissez faire* leader, who regularly verifies his/her sense of inefficacy by avoidance behaviors that may lead to a weakened status within the organization or even demotion. As passive leadership behaviors have poorer work outcomes (Bass, 1999), the negative core self-evaluations of leaders engaging in passive behaviors can easily be reinforced. Active leadership behaviors, on the other hand, may reinforce leaders’ positive evaluations. In line with social exchange theory (Emerson, 1976), positive expectations and subsequent behavioral investments lead to reciprocated loyalty and enhanced efforts on the part of subordinates. The leaders, driven by their positive expectations, display active leadership behaviors such as coaching, supporting, inspiring, stimulating the subordinates, and thereby set positive self-fulfilling prophecies into motion (*Pygmalion effect*; Eden, 1990). When working under active, transformational leadership, the subordinates perform better (Walumbwa et al., 2008), become more innovative (Reuvers et al., 2008), and motivated (Dvir et al., 2002). Consequently, the leader’s positive core other-evaluations get reinforced. Moreover, the success experiences of leaders who perform active, transformational behaviors (Walumbwa et al., 2008; Tsai et al., 2009) may further enhance the leaders’ positive beliefs about the self (James, 1890; Bandura, 1977).

Furthermore, behavior also shapes the leader’s specific evaluations. For example, when a leader avoids a new challenge and starts procrastinating, s/he may immediately feel less in control than before the onset of the procrastination. If the procrastination becomes habitual, it may lead to consolidation of the leader’s negative self-image. Through regularly activating certain specific evaluations by displaying habitual behaviors, leaders further reinforce their core evaluations. And in the self-reinforcing cognitive-behavioral cycle, the fortified beliefs trigger the regular reoccurrence of specific evaluations, behaviors, and situations that are in line with the belief.

#### Core Evaluations and Situation Selection

Another way the cognitive-behavioral system preserves itself can be understood by the concept of situation selection. Situation selection is an effective way of expressing and maintaining one’s personality, by entering situations that are in line with one’s attitudes, motives, and expectations, and avoiding others that contradict them (Emmons and Diener, 1986; Frederickx and Hofmans, 2014). For example, leaders with positive core self-evaluations may be inclined to enter situations in which they are challenged, as they believe that they can successfully cope with the challenges and are inspired by them. Leaders with negative core self-evaluations may be prone to avoid challenging situations that entail a ‘potential for failure’ (Judge et al., 2000, p. 238). However, by avoiding such situations they cannot collect counter-evidence for their negative beliefs. By preventing the disconfirmation of their own fears (Wells et al., 1996), they sustain the coherence of their belief system. Avoidance behaviors are often aimed at preventing the painful feeling that follows the activation of a negative belief; nevertheless, they confirm such beliefs (Young et al., 2003).

### Stable but Not Static: Dynamic System

Beside its inclination to preserve its internal coherence, just like any other organic system, the leaders’ cognitive-behavioral system is also capable of change and reformation. New situations, new appraisals, and new behaviors – if rehearsed repeatedly – may lead to (slow-paced) change in the deep cognitive structures. For example, even though a person’s appraisals of other people’s reliability are partially determined by the person’s a priori expectations, new experiences have the potential of modifying such expectations, in the event that they strongly contradict them (Kramer, 1999). Such new experiences can be triggered by a major change that occurs in the individual’s environment. Within the new circumstances, the features of frequently arising situations alter, the new features trigger new specific evaluations, and these specific evaluations call for novel behaviors. Such changes in the environment can be, for instance, a new position with entirely different tasks that fit the leader’s talents a lot better (or worse) than the previous position; or a new, outstandingly supportive (or hostile) work environment in comparison to the previous workplace. These changes will lead to new, different day-to-day experiences that can transform a leader’s core evaluations. These changes happen slowly and gradually, as described by the sociogenomic personality school in its suggestion that states which are experienced continuously over long time periods cause changes in the neuroanatomical structures of the brain and lead to the modification of traits (Roberts and Jackson, 2008). Another pathway of change in core evaluations may originate within the leader. This route is paved with the leaders’ attempts to change her/his behaviors, appraisals and beliefs, possibly emerging from the recognition that the old cognitive and behavioral patterns are no longer helpful or functional. Repeated challenging and conscious amending of a person’s appraisals *and* behaviors (often guided by coaching, training, or therapy) can slowly modify the deep, cognitive structures (Beck, 1964, 1972, 1991; Felmingham et al., 2007).

### Stability and Dynamism

In sum, leaders’ core evaluations may be maintained *or* modified (1) by being exposed frequently to certain situational features; (2) by repeated specific evaluations; or (3) by repeated leadership behavior. Whether the core evaluations are reinforced or adapted depends on whether the situational features, the specific evaluations, and the behaviors are in concordance or contradiction with the leader’s core evaluations.

Proposition 10: Situational features to which the leader is frequently exposed shape the leader’s core evaluations over the long term, such that (1) when the two are aligned (e.g., supportive organization and positive core evaluations), frequently experienced situations reinforce core evaluations, and (2) when the two are non-aligned (e.g., hostile organization and positive core evaluations), situations may modify core evaluations. (**Figure 1**, arrow 5).
Proposition 11: The more positive a leader’s core evaluations are, the more likely s/he will enter – socially or intellectually – challenging situations (while the more negative the leaders’ core evaluations are, the more likely s/he will avoid such situations; **Figure 1**, arrow 6).
Proposition 12: The leader’s frequently repeated specific evaluations shape the leader’s core evaluations over the long term, such that (1) when the two are aligned, specific evaluations reinforce core evaluations, and (2) when the two are non-aligned, specific evaluations may modify core evaluations (**Figure 1**, arrow 9).
Proposition 13: Situations shape the leader’s core evaluations by (re)shaping the leader’s specific evaluations (**Figure 1**, arrows 7–9).
Proposition 14: The leader’s recurrent behaviors shape the leader’s core evaluations over the long term, such that (1) when the two are aligned with each other (positive core evaluations and active leadership behaviors, or negative core evaluations and passive behaviors), behaviors reinforce core evaluations, and (2) when the two are non-aligned, behaviors may modify core evaluations (**Figure 1**, arrow 10).
Proposition 15: The leader’s behavior shapes the leader’s core evaluations through (1) modifying the situational features (**Figure 1**, arrows 5 and 12), and (2) modifying the leader’s specific evaluations (**Figure 1**, arrows 9 and 11).

## Discussion

What makes some leaders inclined to act in active ways and others in passive ways, and what makes someone an active leader in one situation and a passive one in another? In this article we build on core evaluations theory (Judge et al., 1997) and argue that in any leadership situation entered, leaders assess (1) their own capacities to cope with the task and the interpersonal demands of the situation; (2) the competence and willingness of their subordinate(s) to perform their tasks; and (3) the benevolence, fairness, and dangerousness of the context. Based on the results of their evaluations, they engage in a given leadership behavior (obviously, in most cases this process is swift, automatic, and unconscious).

We suggest that the more the leader perceives him/herself to be able to cope with challenges, others to be trustworthy and the environment to be safe and reliable, the more active behaviors s/he will pursue. If a leader feels insecure or threatened, to prevent feared events from happening s/he may avoid challenges and engage in highly passive leadership behaviors, such as dodging leadership responsibilities altogether (laissez faire) or at least until there is a crisis (passive management by exception). Another response to a low sense of confidence in the self or others may be the close monitoring of events and hyper-vigilance (active management by exception), as an attempt at taking control over the seemingly threatening, uncooperative or inept environment. A moderate level of confidence in the internal and external world is sufficient for the emergence of moderately active behaviors, such as identifying objectives and targets, combined with behaviors that nevertheless still serve the function of mildly controlling people and events, such as rewarding and following up on subordinates (contingent reward). Finally, only a strong sense of efficacy, control and confidence regarding the self, other people and the external world offer the psychological resources that are necessary for the emergence of highly active leadership behaviors, such as demonstrating competence, thinking innovatively, elevating expectations and standards, being inspirational and challenging and stimulating others (transformational behaviors).

In conclusion, we suggest that the more a leader evaluates him/herself, others, and the environment positively, the more s/he will be in possession of the basic psychological resources that are required to engage in complex, (pro)active leading behaviors (see conservation of resources theory, Hobfoll, 1989). Evaluating one or more of the three factors (self, others, environment) negatively triggers less active, ‘safety’ behaviors, such as avoidance or monitoring and controlling subordinates. Thus, leaders’ fleeting evaluations of themselves, others, and the context (their specific evaluations) shape how they act in any given situation and therefore explain within-person fluctuations in leadership behavior. Such momentary evaluations will be partially predicted by the features of the situation at hand. Furthermore, they will be influenced by the leaders’ core evaluations, that is, their inherent tendency to have a generalized high or low opinion of themselves, other people, and their environment. These core evaluations work as filters when perceiving and categorizing information, inclining the individual to make appraisals in line with the core evaluations, and to act accordingly. Thus, core evaluations explain the leaders’ propensity to engage in a certain kind of leadership behavior. We suggest that leaders who have the tendency to evaluate themselves, others, and their environment positively will have a sustained inclination to be active leaders.

## Implications for Leadership Research and Limitations

Because our propositions pertain to both the between- (i.e., core evaluations) and within-leader level (i.e., specific evaluations), studies that go beyond the typical cross-sectional between-subjects design are needed in order to test them. Examples of such designs are daily diary studies (Bolger et al., 2003), in which leaders can be asked about their leadership behavior, the circumstances, and their core self-, other-, and world-evaluations on a day-to-day basis. Another alternative is to conduct experience sampling studies (e.g., Nielsen and Cleal, 2011) where leaders can be asked to rate their leadership behavior, the circumstances, and their core self-, other-, and world-evaluations at random moments throughout their working life. Whereas such designs are harder to implement than the traditional, cross-sectional, between-subjects designs because they, among other things, place considerable demands on the participants, they yield valuable information about within-person changes in leadership behavior and the core evaluations; and this information is necessary to test our propositions. Moreover, such designs allow for the analysis of time-lagged effects, which allows testing the directionality of the proposed relationships.

What complicates the empirical study of our propositions is that measures for core other evaluations and core world evaluations are presently missing (a measure for core self evaluations exists; see Judge et al., 2003). Therefore, there is a need for the development of such instruments. When doing so, it may also be fruitful to identify the overlaps and correlations between the three core evaluations. We expect that negative core evaluations are interconnected (Packer, 1985; Beck, 2011), that is, the lack of trust in others (negative core other-evaluations) will positively correlate with beliefs about the world being a malevolent, unfair, and dangerous place (negative core world-evaluations). Furthermore, seeing the self as helpless, vulnerable, and unable to cope (negative core self-evaluations) will overlap with perceiving the world as a dangerous, unfair, and malevolent place and with seeing other people as untrustworthy. We expect these correlations to exist both on the trait and state level (and between the three positive core evaluations too).

Finally, our model evidently cannot fully explain the emergence of active and passive leadership behaviors. Even though positive beliefs and positive appraisals are prerequisites for active leadership, they cannot entirely predict it. For example, research has shown that affective antecedents have a strong influence on the emergence of transformational and charismatic behaviors (e.g., Walter and Bruch, 2007, 2009; Seo et al., 2008). Leaders must also be in possession of a repertoire of active leadership behavioral scripts to be able to respond to situations with such behaviors. For adaptive behavioral responses to emerge, the accurate appraisal of the situation is necessary but not sufficient, insofar as the individual does not possess a rich behavioral arsenal (Eaton et al., 2009). Furthermore, active leadership behaviors are not equally beneficial in all circumstances. For example, research has shown that transformational leadership behaviors are less beneficial in conditions of high stress (Seltzer et al., 1989), in projects that don’t require the generation of new knowledge (Keller, 2006), and in relation to subordinates with an individualistic mindset (Jung and Avolio, 1999). Therefore, in certain contexts the versatile leader may favor transactional behaviors over transformational ones, based on the consideration that the aforementioned behaviors will be more efficient (in line with contingency theories, e.g., Hersey and Blanchard, 1969). Our model, therefore, aims to provide a basic framework with wide applicability, but cannot alone explain leadership tendencies, and thus intends to complement other approaches that explain the emergence of active and passive leadership.

## Conflict of Interest Statement

The authors declare that the research was conducted in the absence of any commercial or financial relationships that could be construed as a potential conflict of interest.
